# Protecting child health and nutrition status with ready-to-use food in addition to food assistance in urban Chad: a cost-effectiveness analysis

**DOI:** 10.1186/1478-7547-11-27

**Published:** 2013-11-09

**Authors:** Chloe Puett, Cécile Salpéteur, Elisabeth Lacroix, Freddy Houngbé, Myriam Aït-Aïssa, Anne-Dominique Israël

**Affiliations:** 1 Action Against Hunger, 247 West 37th Street, New York, NY 10018, USA; 2 Action contre la Faim - France, 4 rue Niepce, 75 662, Paris, Cedex 14, France; 3 Action contre la Faim - France, 78-D Thanlwin Road, Bahan Township, Yangon, Myanmar; 4 Action contre la Faim - France, Ouagadougou 06 BP 10221, Burkina Faso

**Keywords:** Prevention of acute malnutrition, Prevention of morbidity, Lipid nutrient supplements, Ready-to-use supplementary food, Cost-effectiveness, General food distributions, Supplementary feeding programs, Activity based cost analysis, Chad

## Abstract

**Background:**

Despite growing interest in use of lipid nutrient supplements for preventing child malnutrition and morbidity, there is inconclusive evidence on the effectiveness, and no evidence on the cost-effectiveness of this strategy.

**Methods:**

A cost effectiveness analysis was conducted comparing costs and outcomes of two arms of a cluster randomized controlled trial implemented in eastern Chad during the 2010 hunger gap by Action contre la Faim France and Ghent University. This trial assessed the effect on child malnutrition and morbidity of a 5-month general distribution of staple rations, or staple rations plus a ready-to-use supplementary food (RUSF). RUSF was distributed to households with a child aged 6–36 months who was not acutely malnourished (weight-for-height > = 80% of the NCHS reference median, and absence of bilateral pitting edema), to prevent acute malnutrition in these children. While the addition of RUSF to a staple ration did not result in significant reduction in wasting rates, cost-effectiveness was assessed using successful secondary outcomes of cases of diarrhea and anemia (hemoglobin <110 g/L) averted among children receiving RUSF.

Total costs of the program and incremental costs of RUSF and related management and logistics were estimated using accounting records and key informant interviews, and include costs to institutions and communities. An activity-based costing methodology was applied and incremental costs were calculated per episode of diarrhea and case of anemia averted.

**Results:**

Adding RUSF to a general food distribution increased total costs by 23%, resulting in an additional cost per child of 374 EUR, and an incremental cost per episode of diarrhea averted of 1,083 EUR and per case of anemia averted of 3,627 EUR.

**Conclusions:**

Adding RUSF to a staple ration was less cost-effective than other standard intervention options for averting diarrhea and anemia. This strategy holds potential to address a broad array of health and nutrition outcomes in emergency settings where infrastructure is weak and other intervention options are infeasible in the short-term. However, further research is needed to establish the contexts in which RUSF is most effective and cost-effective in preventing acute malnutrition and morbidity among vulnerable children, compared to other options.

## Background

Use of therapeutic food products, in the form of Lipid Nutrient Supplements (LNS), has been well-documented as a proven method to treat severe acute malnutrition (wasting) in children [1-3]. Recently there has been growing interest in the potential of LNS for preventive benefits on child nutrition and health outcomes when used as a complementary food or a micronutrient-rich food supplement for young children, particularly in the context of humanitarian emergencies [4]. Several studies have assessed the effect on these outcomes of LNS in both supplementary and therapeutic form, i.e. ready-to-use supplementary foods (RUSF) and ready-to-use therapeutic foods (RUTF).

Evidence is inconclusive on the effectiveness of fortified foods (including LNS) in preventing deterioration of child health and nutrition status. A systematic review of effectiveness of complementary feeding interventions on growth, morbidity and child development outcomes, found results to be inconsistent, context-specific, and dependent on the quality of program design and implementation [5].

Two studies from the Maradi region in Niger found a significant protective effect of LNS on child wasting status [6,7]. Compared to other commonly-used supplementary foods such as corn soy blend (CSB), LNS has supported higher weight gain and has recovered children from moderate wasting [8,9] or prevented the onset of wasting among non-malnourished children [10] in some studies. Other research has shown equivocal evidence of the superiority of LNS to CSB in prevention [11] and treatment [12-14] of moderate wasting.

Evidence for the effect of LNS on linear growth is also varied. Some research has found that LNS supplementation boosts linear growth [15-18], particularly among children from more disadvantaged households [19]. Other studies found LNS to have no effect or limited effect in this regard, assessed either compared to a control group [20] or to CSB [13,21,22]. One trial found that differences in weight and length gain between infants receiving LNS and those receiving no supplement were comparable with gains achieved using fortified blended flours such as CSB [14].

Further assessments have compared the effects of LNS with other treatments on common childhood illnesses, including diarrhea, cough, fever and malaria. While several such studies found no difference in morbidity rates attributable to LNS [6,14-17,21], some found beneficial effects on morbidity of RUSF [18] and RUTF [23].

Anemia is another outcome of particular interest, given the multiple micronutrients in LNS. Several studies found significantly improved anemia rates and blood hemoglobin concentration conferred on children receiving LNS supplementation [14,16,18]. Other studies found no effect of LNS on hemoglobin levels or anemia outcomes, when compared to CSB [9,19].

When taken together, this body of research indicates that while LNS does convey some protective benefits to children in vulnerable environments, the extent of these benefits is unclear. Given the lack of conclusive evidence, and the high cost of LNS relative to other options, there is a need for prudent and rational use of these products and for further evidence on the effectiveness and cost-effectiveness in different settings of using such food products in a preventive manner.

This study aimed to provide evidence on the cost-effectiveness of using LNS to protect child health and nutrition outcomes.

In 2010, Action contre la Faim France (ACF-France), an international non-governmental organization, and Ghent University in Belgium implemented an operational research intervention in Abeche in eastern Chad for 5 months (June-October 2010) during the seasonal ‘hunger gap’, to improve household food security and prevent acute malnutrition in children from vulnerable households. A household was considered vulnerable if it either had a household head who was disabled, pregnant or lactating, or had a ratio of economic dependents to working members of 4:1 or greater. Food assistance (FA) in the form of staple rations was distributed to all vulnerable households. Additionally, households with a child aged 6–36 months who was not acutely malnourished (weight-for-height ≥ 80% of the NCHS reference median, and absence of bilateral pitting edema), received an additional supplement of Plumpy’Doz®, a LNS used as Ready-to-Use Supplementary Food (RUSF) and produced by Nutriset (Malaunay, France), to prevent acute malnutrition in these children.

A cluster randomized controlled trial (RCT) was conducted to measure the effect of RUSF on child nutrition and morbidity outcomes, when added to a staple food ration for the household [18]. The effectiveness of both programs (food assistance alone: FA; and FA plus the additional RUSF component: FA + RUSF) was compared in terms of child anthropometry (wasting measured as both low weight-for-height (WHZ) and mid-upper arm circumference (MUAC), and stunting as low height-for-age (HAZ)), morbidity (diarrhea, fever and respiratory tract infection via caregiver recall), and hemoglobin concentration. The RCT found no significant difference between the two groups in terms of wasting incidence, which the researchers presumed could be due to a range of factors including limited statistical power, possibly insufficient daily kilocalorie contribution (approximately 247 kcal per dose) to effect weight gain within the timeframe of the study and potential dilution of RUSF nutrients by the general food distribution [18]. However, other outcomes showed significant improvement among children receiving RUSF. Children in the FA + RUSF group had a small but significant increase in linear growth, and significantly reduced morbidity due to diarrhea (defined as having at least three loose stools within a day via 1-week caregiver recall) and fever (as diagnosed by caregiver via 1-week recall). Further, these children had significantly higher hemoglobin levels and a lower prevalence of total anemia (hemoglobin <110 g/L) at the end of the program.

Since no effect was seen on the primary outcome of wasting, we assessed cost-effectiveness in terms of the secondary outcomes of anemia prevalence and diarrhea incidence. While linear growth outcomes were statistically significant, previous research suggests that the small improvements seen in children receiving FA + RUSF (0.09 cm/month; 0.03 HAZ) may not be biologically significant [24-29]. Therefore, while linear growth improvement was an important program outcome, it was not included in assessment of program cost-effectiveness.

The specific objective of this analysis was to assess the cost-effectiveness of adding RUSF—with its related management and logistics—to food assistance (FA + RUSF), to avert cases of anemia and episodes of diarrhea during the seasonal hunger gap, compared to FA alone. The results contribute evidence of the economic implications of using RUSF in a preventive manner, and can be used to inform future programming.

## Methods

### Description of the context and intervention

Chad is a landlocked country in the Sahelian belt, suffering from an annual hunger gap between June and October. The Ouaddaï region in eastern Chad, with Abeche as the capital city, experiences some of the highest rates of childhood acute malnutrition in the country. This area additionally suffers from a limited number of humanitarian actors, due to ongoing security concerns [30]. In 2010, below-average cereal production and high food prices led to increased food insecurity in eastern Chad [31].

In the first half of 2010, UNICEF reported that an increasing number of areas were affected by SAM, including hundreds of thousands of internally displaced people and refugees in eastern and southern Chad [30]. ACF surveys in Abeche reported that in mid-2009, wasting prevalence was 20.6% with 3.2% severe wasting and in early 2010, wasting prevalence was 16.8% with 2% severe wasting [32,33]. These rates are consistently above the WHO threshold of 15% [34], demonstrating a need for intervention. Moreover, project documents noted that admissions to nearby CMAM (community-based management of acute malnutrition) programs were higher and later in the season than expected given annual trends [35].

Table 1 presents the baseline nutrition status of children participating in the program. While acute malnutrition (in terms of WHZ and MUAC) is not at emergency levels, prevalence of anemia is above 60%. There is no baseline measure of diarrheal morbidity; however a feasibility study conducted by ACF in February 2010 in Abeche indicated that poor sanitation and hygiene, common causes of childhood diarrhea, were serious issues affecting children’s health and nutrition status, particularly in the most vulnerable neighborhoods where the intervention was targeted. Poor sanitation was exacerbated by severe rainfall and flooding in Abeche during July and August of 2010.

**Table 1:** Sample characteristics at baseline

**Characteristic**	**FA alone**	**RUSF component**
MUAC, cm, mean (SD)	14.0 (1.0)	14.0 (1.0)
Wasting, *n* (%)		
WHZ < −2	59 (13.4)	81 (13.6)
WHZ < −3	1 (0.2)	3 (0.5)
Anemia (hemoglobin <110 g/l), *n* (%)	270 (61.5)	370 (61.9)

FA: Food Assistance; RUSF: Ready-to-use Supplementary Food.

Taken from [18].

Program implementation took place in 7 administrative sectors of Abeche, which were divided into 14 geographical clusters. These clusters were then randomized during an official ceremony with community leaders. Seven clusters received only Food Assistance (FA) rations consisting of staple foods (sorghum, legumes, palm oil, sugar and salt, approx. 1,800 kcal [18]) without a fortified blended food such as CSB, and 7 received FA with additional provision of RUSF (FA + RUSF). Operational research was implemented within this structure for 5 months (June-October 2010) with an RCT, with a protocol approved by the Ethics Committee of the University Hospital of Ghent in Belgium and Chadian authorities; study methods have been described elsewhere [18]. Acutely malnourished children were enrolled in the standard protocol for management of acute malnutrition, available at health center level and technically supported by ACF.

Starting 2 months before implementation commenced, a Sensitization Project Manager raised awareness about the program by creating sensitization materials and discussing with community leaders and health officials within the intervention area. This was an important step due to the lack of humanitarian projects in the city, and community suspicion of such projects. Beneficiary selection was conducted with the assistance of community leaders on the basis of ACF’s vulnerability criteria; ACF subsequently verified the final household lists.

Each month, food distributions were conducted at 5 distribution sites accessible to the participating communities. Since communities donated distribution sites, constructions were built temporarily and broken down when distribution was finished at each site. Monthly, distribution took between 7 and 8 working days (over approximately 2 weeks) with additional days required for setting up and breaking down the distribution sites or rebuilding them due to weather damage. During the remainder of each month, program staff were engaged in Food for Training sessions (for the first 2 months only, as described below), raising community awareness about the date of the next distribution, replenishing food rations for the subsequent distribution, entering data and conducting surveys (including a baseline survey, two SMART surveys, and Post Distribution Monitoring). All beneficiaries entered the general distribution site, and were channeled into different “circuits” based on whether their distribution card identified them as being part of the FA or FA + RUSF group. Data on nutrition and morbidity status was collected at distribution sessions: anthropometry was measured monthly, episodes of diarrhea were recalled for the prior week on a monthly basis, and hemoglobin concentration was measured both at baseline (June) and end of intervention (November) or when the child was discharged from the study.

During the first 2 months of implementation, rations were distributed to all beneficiaries (FA and FA + RUSF groups) conditional on attendance at Food for Training sessions on hygiene-related themes. These trainings may have improved beneficiary practice to some extent, however due to a deteriorating security situation in Abeche, these sessions were discontinued and by August the program was limited to unconditional food distribution.

The FA program and the RUSF component shared many activities and resources. Both program components shared the same intervention area (14 geographical clusters) and general program structure. Households targeted for the FA program were also included in the RUSF component if they were located in one of the 7 FA + RUSF clusters, and had a child who fit the admission criteria. The FA program consisted of 5 monthly distribution sessions; at these same sessions, RUSF was distributed to qualified households.

In terms of staff, each program component had its own dedicated personnel, including a Head of Project (along with an assistant in the RUSF component) who was responsible for managing and overseeing activities within their program component. Additionally there were some shared staff who implemented activities common to both interventions; these included supervisors, distribution monitors and a community mobilizer who sensitized the community about the entire program.

While the RUSF component benefited from sharing some of the infrastructure of the FA program, given its different focus it also required separate trainings for dedicated staff, and additional supplies (e.g. MUAC strips, sensitization materials for beneficiaries receiving RUSF, etc.). Other resources were employed specifically for the RUSF component, related to anthropometric measurement, including staff and equipment, and several surveys.

The RUSF component of the program was directly related to the operational research; therefore many program staff spent a portion of their time on research-related activities. Time spent on research was estimated and excluded so that this analysis would only account for time spent implementing the project.

### Analytical strategy

This study assessed the incremental cost-effectiveness of the addition of RUSF to a FA ration, for improving selected child health and nutrition outcomes. Both total FA program costs and incremental costs of the RUSF component were estimated from the societal perspective, thereby including all costs related to program implementation and participation incurred by institutions and communities. The World Food Program (WFP) provided food rations and support for the food distribution sessions. All other institutional costs were covered by ACF. Costs were calculated with a combination of accounting records and “ingredients” estimates using unit costs and quantities of inputs [36].

Activity-based costing (ABC) is a method of cost categorization and analysis in which all costs of a program are allocated to its activities [36,37]. Traditional accounting cost centers organize costs by input category (e.g. personnel, medical supplies, capital costs) [36]. Activity-based costing takes analysis of costs one step further by allocating these input costs to activity-based cost centers, representing the activities for which the input was used. This enables assessment of the specific resource use of various program activities.

An ABC methodology was applied to all costs in this analysis, including both accounting costs and ingredients estimates; this methodology was used to achieve multiple analytical goals. First, the ABC methodology assisted in separating all program costs into FA and RUSF program components, thereby facilitating an analysis of incremental costs of the RUSF component. Second, the ABC method was used to allocate costs of all inputs that were shared between the FA and RUSF program components, and for which the allocation was not straightforward (i.e. staff, vehicles, office running costs). Application of the ABC methodology will be further described in the following sections.

Effectiveness data was taken from the operational research study connected to the program [18]. Incremental costs were calculated per case of anemia averted and episode of diarrhea per child-month.

### Data collection

Data was collected between February and August 2012 by reviewing reports and financial documents, and conducting key informant interviews via teleconference with primary implementing staff including heads of project, program managers and technical coordinators (n = 13).

### Cost estimation

#### Institutional costs

Costs of inputs used in the program, which were not recorded in accounting records, were estimated with data from key informant interviews and project documentation. These included cost of storage for FA rations and RUSF, transport of rations, and staff involved in implementation and distribution support but not recorded in program accountancy. WFP provided the cost of staple rations.

#### Community contributions

Communities contributed time and resources to participate in this program. Direct and indirect beneficiary costs were estimated via key informant interviews with implementing staff familiar with local travel distances and transportation prices. Estimates were calculated for differential time spent at monthly distribution sessions by beneficiaries receiving FA and FA + RUSF respectively; and for round-trip travel to and from the distributions. It was assumed that on the way back home from the distribution point, when carrying their FA rations and RUSF, one-half of beneficiaries would take local transportation while the other half would walk home with a family member. The intervention occurred during harvest time, and many beneficiaries (40% according to program documentation and key informants) had to travel from their fields into town for the distributions. It was assumed that these beneficiaries would travel via bus for two hours one-way door-to-door to return to town from their fields.

Cases of moderate anemia identified during the program were referred to the local health clinic for treatment consisting of mebendazole, iron and folic acid supplements. Sixteen out of the 45 children referred actually accepted the referral. It was assumed that accessing this treatment would take a caregiver one-half day of travel and waiting time, that no other direct costs would be incurred, and that all other costs would be covered by the clinic. Estimates do not include any direct or indirect costs incurred by households for provision of additional care after the clinic visit.

Sites for the monthly food distributions were donated by communities (schools, personal yards, stadiums). While private properties were the only suitable distribution sites available, it was difficult for owners to give up this land for all distribution days. Costs for these sites were estimated for all 38 distribution days as the daily rental price for a room used for meetings at a private center in Abeche town (60 EUR), multiplied by 6 to account for the total space needed; this price was discounted by 25%, as a best-guess estimate to account for its being a charitable donation from the community.

Finally, 7 community members per quarter were engaged in a 3-week process to select beneficiaries. Key informants estimated that heads of quarter (n = 2 per quarter) spent 3 weeks full time, while the remaining members of this committee spent 30% of their time on this activity.

Cost of community members’ time was valued in various ways. Beneficiary time was valued using an average agricultural daily wage estimated from previous studies in different African countries and different years (Mali and Zambia: 2006; Ghana and Malawi: 2009) [38-40] at 1.45 EUR per day (0.23 EUR per hour). Community committee members’ time was valued at the daily wage paid by the ACF Abeche mission to casual laborers at 6.10 EUR per 8-hour workday.

### Data analysis

#### Assembling cost data

Accounting data was adjusted to arrive at the final estimates.

Costs for the supplementary food (RUSF) include only the food used during the program. Therefore RUSF shipping costs were adjusted to account for this lower volume. This entailed using a higher international shipping rate than was actually charged to the program to account for the higher cost per unit charged for shipping smaller amounts of product, and a lower cost for truck rental and fuel for local shipping. The potential cost savings of using locally produced RUSF were explored during sensitivity analysis.

The program incurred many research costs related to the RCT, which were excluded as they did not contribute to program outcomes. Baseline and SMART surveys produced information that informed implementation, therefore costs for these surveys were included. Costs of routine program monitoring were included. To reflect costs of a more typical program scenario, one-half of some research-related costs were included as program costs, e.g. double measurement of anthropometric indicators taken to ensure data quality for the RCT, including cost of anthropometrists, data entry clerks, wooden measuring boards, scales and batteries for scales.

Food for Training activities were initiated but not completed. The cost of these activities was included assuming that even limited exposure to training could influence beneficiary behavior and therefore program outcomes.

Cost of capital items was amortized using standard tables (3 years for computers, 5 years for other equipment), discounted at a rate of 3%, and one year’s value was included, given the program’s short duration.

Costs from the accounting system were regularly converted to Euros from Central African Francs using official bank rates, and were not adjusted for inflation as they covered less than one year. All costs are presented in 2010 EUR.

#### Applying activity-based costing in cost allocation

The ABC methodology was applied to all costs, including both accounting costs and ingredients estimates, and costs to both institutions and communities. During the ABC analysis, all costs were considered as “inputs” required when implementing a particular activity.

Activity-based cost centers in this analysis represent the overarching program components (FA and RUSF) which are comprised of both component activities (e.g. distributions, sensitization, training) and the inputs required for activities in each cost center (e.g. personnel, staple rations, distribution sites).

The ABC method assisted in isolating costs of the RUSF component from costs of the overarching FA program, enabling an analysis of incremental costs of the RUSF component. First, costs were allocated to the FA or RUSF cost centers based on direct utilization where possible (i.e. the cost of RUSF was allocated to the RUSF cost center). Second, shared costs, such as management, shared equipment and other program support, for which allocation was not straightforward, were allocated to cost centers separately using the activity time allocation of staff. Staff people implement programs, and their time allocation to various activities within a program is therefore intended to represent the relative “resource-intensiveness” of each activity. For example, if a program with 2 activities has a staff time allocation of 50% to each activity, then each activity requires an equal amount of staff time and, therefore, of resources for program support. Support costs included in this analysis represent those contained within the ACF accounting records for only this program, and therefore do not include costs related to supporting other programs implemented by ACF concurrently.

Key informant interviews were conducted with key implementing staff, along with management and coordination staff involved with the program, to determine the proportion of their time devoted to either the FA program or to the RUSF component and its related management and logistics. Time dedicated to research activities was excluded, and a scale factor was applied to these estimates so proportions summed to 100%. An average time allocation proportion was then calculated for each activity, to reflect the overall staff time proportion required for each activity. The effect of these proportions of support costs on relative cost-effectiveness of FA versus RUSF was explored during sensitivity analysis.

#### Cost analysis

Ingredient estimates were entered and analyzed, and accounting data adjusted and allocated, using Microsoft Excel software [41]. Costs were analyzed first in terms of input category, using those categories indicated in the accounting records, and then by allocating these inputs to the activity-based cost centers.

#### Cost-effectiveness analysis

Cost per child was calculated using the number of children included in the program at baseline, regardless of outcome. This included 1,071 children total, a subset of whom received only FA (n = 458) and another subset who received FA + RUSF (n = 613). Cost per child of FA alone was calculated by dividing total costs of the FA program by all participating children (n = 1,071). An incremental cost per child was calculated by dividing the incremental cost of the RUSF component by the number of children receiving RUSF (n = 613).

Incremental cost-effectiveness ratios (ICERs) represent the additional cost per improved outcome achieved by the RUSF component compared to FA alone. ICERs were calculated by dividing the cost per child (calculated as total cost of program/number children in program) by the difference in morbidity outcomes (i.e. cases of anemia or episodes of diarrhea per child-month in FA or FA + RUSF/number of children in FA or FA + RUSF). Since both numerator and denominator of the ICER are divided by the number of children in the program, the ICERs represent the cost per case of child morbidity averted.

Costs and effects were modeled with TreeAge Pro 2012 software [42], using a decision tree with two branches: one for the FA program, and one for the additional FA + RUSF component. The model assessed the incremental cost per case of anemia averted and episode of diarrhea per child-month averted in the FA + RUSF program area compared to FA alone, assuming various levels of “willingness to pay” to achieve these outcomes. Willingness to pay refers to the value of the ICER that society would consider acceptable to achieve program outcomes [43].

Sensitivity analyses were conducted to determine whether significant changes would occur in the base case ICER estimates, given changes in various parameters of interest. In the first phase, univariate sensitivity analyses were conducted for individual variables, representing both costs and effects, over a plausible range of values. In a second phase, probabilistic sensitivity analyses were conducted with 100,000 replicates per analysis to assess variation in multiple variables simultaneously.

During sensitivity analyses, best and worst case scenarios were modeled using a range of +/− 25% on base case observations. For the diarrhea episode outcome, an additional 10 percentage points were added to the variance in the FA group to account for a loss to follow-up that was 10% higher over the course of the study than the FA + RUSF group. Diarrhea episode data was collected on a monthly basis; the loss to follow-up affects monthly incidence data, but not anemia prevalence data, which was collected at the end of the program. Loss to follow-up was experienced in the first follow-up measurement and was corrected by the third follow-up after caregivers in the FA group were given incentives (e.g. laundry soap, mosquito nets) for bringing their children to distribution sessions.

For one-way sensitivity analyses, the incremental cost per child of the RUSF program component was broken down into several elements to model various scenarios. To test the potential of locally-produced RUSF to improve cost-effectiveness, a scenario was modeled using available data on price differentials between imported and locally produced products. The “worst case” scenario used estimates from the base case analysis of RUSF imported from Nutriset (2.83 EUR/kg) and all international and local shipping costs included from the program accounting records. The best case scenario was modeled using published data from Malawi [44], suggesting that local product would cost 1.89 EUR/kg (adjusted for inflation and currency exchange), representing a 33.2% decrease from the base case. This estimate includes duty and shipping for imported ingredients (multivitamin mix), however it may not include other costs of local production such as quality assurance efforts for aflatoxin testing, etc., and may therefore underestimate the real costs of local production. An additional scenario was modeled using a cost estimate from a local RUSF producer in Niger of 3.39 EUR/kg (Nutriset, communication to ACF), representing a 19.8% increase from the base case. For these modeled scenarios, only the difference in purchase price of local product was used; it was assumed that the local product was available in Chad, and so local shipping costs were assumed to be the same as in the base case, with no international shipping costs incurred.

Support costs allocated to the program were varied over a plausible range. The base case included 35% of support costs, based on percent of staff time allocation to the RUSF program component, in accordance with the activity-based costing methodology. The worst case scenario included 50% of support costs while the best case scenario included 15% of support costs, as an assumed plausible range.

All other program costs were modeled using a range of +/− 25% on base case observations.

## Results

### Cost outcomes

Costs were assessed first by input categories, and then by allocating these inputs to activity categories (i.e. activity-based cost centers).

#### Input cost shares

**Figure 1:**
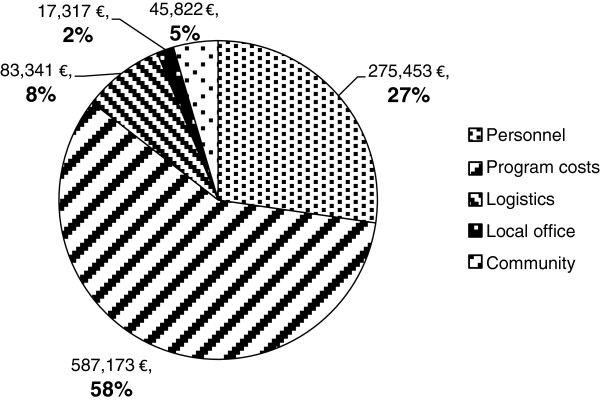
Cost per input category (EUR).

Figure 1 shows total program resources by input categories.

Personnel expenses represent about one-third of total costs (27%). Twenty-nine percent of these human resource expenditures were allocated to technical staff and 71% to support staff. Additionally, 66% of personnel costs were for Expatriate staff and 34% for National staff.

Logistics comprised 8% of costs, including transportation, storage and communications. Local office costs make up the smallest proportion of total costs at 2%, and represent overhead costs which are shared among programs, including rent, computer equipment, stationery, and maintenance. Program costs made up 58% of expenses, and community contributions made up 5% of total resource use. These will be described in greater detail in the following sections.

#### Activity-based cost centers

Two activity-based cost centers were derived: (1) the Food Assistance (FA) program, and (2) the additional activities related to RUSF distribution and management. Program costs were allocated to these two main activities based on direct utilization where possible. Shared costs, such as management and other program support, were allocated via the activity-based costing methodology, using time allocation proportions of implementing staff. Costs related to community sensitization were assigned to FA (25%) or RUSF (75%) according to findings from staff time allocation interviews.

Excluding time dedicated to research activities, implementing staff reported an aggregate of 65% of time dedicated to the Food Aid program and 35% to the RUSF component. These results did not change significantly when including or excluding the time of management and coordination staff, after adjusting for research time allocation. Descriptions of cost centers, including their component activities, inputs, and data sources are presented in Table 2.

**Table 2:** Description of cost centers and data sources

**Cost Center Description**	**Component Activities**	**Inputs Required per Cost Center**	**Data Sources**
Food Assistance (FA):	Monthly distributions of staple food rations	Personnel devoted to FA-related activities (or % staff time)	Review of financial and program documentation. Key informant interviews with administrative staff. Time allocation interviews with implementing staff.
*All costs related to monthly distribution of staple rations and activities supporting these distributions*			
	Food for Training activities	Staple rations + storage & transport	
	Post-distribution monitoring	Value of beneficiary time spent traveling to, waiting for and attending (FA group) distributions	
	Sensitization related to FA component	Value of beneficiary time traveling to and waiting at clinic for anemia treatment	
	Staff training	Value of distribution sites	
		Value of time spent by community leaders in beneficiary selection	
		Equipment & Supplies, including materials for distributing rations and building distribution sites	
		Program support costs	
RUSF component:	Distribution of RUSF at monthly distribution sessions	Personnel devoted to additional activities related to RUSF (or % staff time), and its management and logistics	Review of financial and program documentation. Key informant interviews with administrative staff. Time allocation interviews with implementing staff.
*All costs related to addition of RUSF to monthly distributions, and related management and logistics*			
	Nutrition surveys	RUSF sachets used in program + storage & transport	
	Sensitization related to RUSF component	Value of additional time spent by beneficiaries (FA+RUSF group) attending distributions	
	Staff training	Equipment & Supplies, including medical & anthropometric equipment	
		Program support costs	

RUSF: Ready-to-use Supplementary Food.

#### Activity cost allocation

Table 3 presents costs allocated from the various program inputs to each activity-based cost center.

**Table 3:** Cost per cost center

**Inputs allocated per cost center**	**Costs (€)**	**% of FA or RUSF costs**^**†**^	**% of Total costs**
**Food Assistance (FA)**			
*Institutional costs:*			
**Personnel**	90,017		
Expatriate Technical Staff	*56,055*	7.19	5.55
Local Technical Staff	*33,961*	4.35	3.37
**Program costs**	525,622		
Staple rations	*498,029*	63.84	49.35
Distribution materials	*24,459*	3.14	2.42
Food for Training activities	*2,492*	0.32	0.25
Sensitization	*642*	0.08	0.06
**Logistics**	9,128		
Ration transport ǂ	*4,628*	0.59	0.46
Ration storage	*4,500*	0.58	0.45
Support costs* allocated	109,837	14.08	10.88
Subtotal institutional (% FA total)	**734,604**	**94.2%**	
*Community contributions:*			
Beneficiary time & transportation	32,876	4.21	3.26
Value of loaned sites for distribution days	10,260	1.32	1.02
Leaders’ time in beneficiary selection	2,349	0.30	0.23
Subtotal community (% FA total)	**45,485**	**5.8%**	
**Total cost of FA (% total)**	**780,089 €**	**100%**	**77%**
**+ RUSF component**			
*Institutional costs:*			
**Personnel**	84,821		
Expatriate Technical Staff	*62,956*	27.49	6.24
Local Technical Staff	*21,865*	9.55	2.17
**Program costs**	61,551		
Incentives for caregivers^¥^	*19,909*	8.69	1.97
Medical & anthropometric equipment ^Ω^	*15,283*	6.67	1.51
RUSF	*7,483*	3.27	0.74
Surveys	*7,376*	3.22	0.73
Other assorted supplies	*7,276*	3.18	0.72
Sensitization	*2,179*	0.95	0.22
Daily laborers for distributions	*1,366*	0.60	0.14
Training	*679*	0.30	0.07
**Logistics**	22,636		
RUSF transport ǂ			
International air transport	*15,915*	6.95	1.58
Local vehicle transport	*5,067*	2.21	0.50
RUSF storage ǂ	*1,655*	0.72	0.16
Support costs* allocated	59,671	26.06	5.91
Subtotal institutional (% RUSF total)	**228,679**	**99.9%**	
*Community contributions:*			
Beneficiary time & transportation (additional)	338	0.15	0.03
Subtotal community (% RUSF total)	**338**	**0.1%**	
**Incremental cost of RUSF component (% total)**	**229,017 €**	**100%**	**23%**
**Total costs (FA + RUSF)**	**1,009,106 €**		**100%**
Institutional costs	963,283		95%
Community contributions	45,822		5%

Costs and percentages may not match added totals due to rounding.

FA: Food Assistance; RUSF: Ready-to-use Supplementary Food.

^†^This column indicates the proportion of costs for either the FA or RUSF program component; proportion of total program costs (FA + RUSF) is indicated in the last column.

^¥^ These were items given to caregivers, including mosquito nets and laundry soap, to encourage them to attend distributions.

^Ω^ These include minimal costs incurred for mebendazole, iron and folic acid tablets used in managing cases of moderate anemia.

ǂ These costs included related staff such as personnel, guards, and daily workers to unload rations (in the case of FA).

* Support costs include costs for management and other program support services which are shared among program activities, including inputs such as personnel, logistics costs such as vehicles and communications, local office running costs, and supplies such as stationery. These costs were allocated to each program component using an activity-based costing methodology.

#### Cost center comparison

The Food Assistance (FA) cost center represented 77% of total program costs (780,089 EUR), and costs related to the incremental RUSF component made up the remaining 23% (229,017 EUR), with a total program cost of 1,009,106 EUR. The largest program expense was the staple rations, at 49.35% of total costs. When combined with the cost of RUSF (0.74% of total costs), one-half of total program costs were attributable to food items.

In the FA program, materials used for monthly distribution sessions in the 7 selected quarters of Abeche comprised 2.42% of total costs and included supplies (i.e. buckets, plastic sacks for ration distribution), printing of identification cards, and other assorted stationery and equipment. Ration transport and storage together represented just under 1% of total costs (including fuel, drivers and guards). Less than 1% (0.25%) of costs were related to the partially-implemented Food for Training activities conducted with beneficiaries for the first 2 months of the program on health and hygiene topics. Sensitization represents a small proportion (0.08%) of FA costs, incurred in sensitizing and informing the community and local leaders about the FA program. Support costs allocated to the FA program represent 14% of FA costs and 11% of total costs.

For the RUSF program component, technical expatriate staff represented 27.49% of the costs for the RUSF component alone, with an additional 9.55% for local technical staff; this indicates that a significant proportion of additional resources required for this program component were for technical support. The RUSF itself, costs of which were adjusted for quantities used in the program (as opposed to total quantity originally ordered), represents only 0.74% of total program costs. Combined, local and international transport of RUSF represent 2.08% of total costs, and its local storage comprises an additional 0.16% of total costs. Other assorted supplies, comprising less than 1% of total costs, included stationery and field costs of nutrition survey teams. Incentives for caregivers (e.g. mosquito nets, laundry soap) comprised 1.97% of total costs. Medical and anthropometric equipment (including scales, batteries, measuring boards, medicines, etc.) made up 1.51% of total costs. Costs for baseline and SMART surveys represent 0.73% of total costs. Sensitization comprised a small proportion of RUSF-related costs (0.22%) incurred in informing the community and local leaders about the RUSF component of the program. Costs related to RUSF-related staff training comprised 0.07% of total costs.

Community costs represented 5% of total resource use. While this may indicate that the time and cost involved in participating in this food distribution were not a major constraint to community access and utilization of the program, there are at least two possible caveats to this assumption. First, the high loss to follow-up, among children receiving FA alone, in the first two months before additional incentives were offered indicates that communities felt it was not worthwhile to attend distributions with their child without provision of a supplementary food like RUSF. Second, beneficiaries engaged in planting and harvesting field crops had difficulty attending the distributions, which required an additional trip into town.

### Cost-effectiveness outcomes

#### Base-case analysis

Base case cost-effectiveness results are presented in Table 4. The cost per child of FA alone was 728 EUR when including all children participating in the FA program (n = 1,071); when including only those children receiving RUSF in addition to FA (n = 613), the incremental cost per child for the RUSF component was 374 EUR. A total cost for children receiving both FA and RUSF can be calculated by adding cost per child of FA and RUSF (1,102 EUR).

**Table 4:** Base case cost-effectiveness results

**Outcome**	**FA alone**	**RUSF component**
Total cost (EUR)	780,089	229,017
# children* in program	1,071	613
Total cost per child of FA alone* (EUR)	728	--
Incremental cost per child receiving RUSF* (EUR)	--	374
Episodes of diarrhea per child-month	1.17	0.81
Anemia prevalence	66.8%	56.5%
*Diarrhea outcome:*		
Incremental cost† (EUR)	--	374
Incremental effectiveness	--	0.36
ICER (€/episode of diarrhea averted per child-month)	--	1,038
*Anemia outcome:*		
Incremental cost (EUR)	--	374
Incremental effectiveness	--	10.3%
ICER (€/case of anemia averted)	--	3,627

FA: Food Assistance; RUSF: Ready-to-use Supplementary Food; ICER: Incremental cost-effectiveness ratio

* Cost per child was calculated using the number of children included in the program at baseline, regardless of their outcome. The number of children in both programs was different: all children participated in the FA program (n = 1,071), a subset of these children received only FA (n = 458), another subset received RUSF in addition to FA (n = 613).

In the FA + RUSF intervention, compared to FA alone, the incremental cost was 1,038 EUR for each additional episode of diarrhea averted, and 3,627 EUR for each additional case of anemia averted.

### Sensitivity analysis on variable parameters

Sensitivity analyses were conducted to determine whether plausible variation in study parameters significantly changed study outcomes. Table 5 presents variable parameters and ranges used in sensitivity analyses.

**Table 5:** Model input parameter values and ranges

**Parameter**	**Base case**	**Worst case**	**Best case**	**Source of base case (and range)**
*Effectiveness measures:*				
Intervention area: diarrhea episodes per child-month	0.81	1.01	0.61	Base case: [18]
				Worst case: + 25% of the base case
				Best case: - 25% of the base case
Control area: diarrhea episodes per child-month	1.17	1.58	0.76	Base case: [18]
				Worst case: + 35% of the base case*
				Best case: -35% of the base case*
Intervention area: anemia prevalence	56.50 %	70.63 %	42.38 %	Base case: [18]
				Worst case: + 25% of the base case
				Best case: -25% of the base case
Control area: anemia prevalence	66.80 %	83.50 %	50.10 %	Base cas: [18]
				Worst case: + 25% of the base case
				Best case: - 25% of the base case
*Incremental cost per child of RUSF component (EUR):*				
Incremental cost	374	484	249	Base case: Total program costs (Table 3)
				Worst case: + 25% of all program costs + 50% of support costs
				Best case: -25% of all program costs + 15% of support costs
*Component costs*: ^ǂ^				
RUSF and related shipping	38	38	8	Worst case: Imported RUSF scenario: from program accounting
				Best case: Local production scenario: reduced RUSF costs [44] and no international shipping
Other program costs	238	298	179	Base case: Program accounting
				Worst case: + 25% of the base case
				Best case: -25% of the base case
Support costs allocated	97	138	41	Base case: 35% Staff time allocation
				Worst case: 50% allocation
				Best case: 15% allocation

RUSF: Ready-to-use Supplementary Food

Costs have been rounded to the nearest euro.

* Estimates for diarrhea incidence in the control area are given a wider range due to higher loss to follow-up on monthly incidence outcomes in the control area, and resulting higher uncertainty in these estimates.

ǂ Component costs are only used in univariate sensitivity analysis.

#### Univariate sensitivity analysis

In the tornado diagrams below, the horizontal axis represents the range of ICER values occurring when each parameter listed in the figure was varied individually. These parameters include incremental costs of the RUSF component, and effectiveness of both FA and FA + RUSF. The vertical line represents the base case ICER.

**Figure 2:**
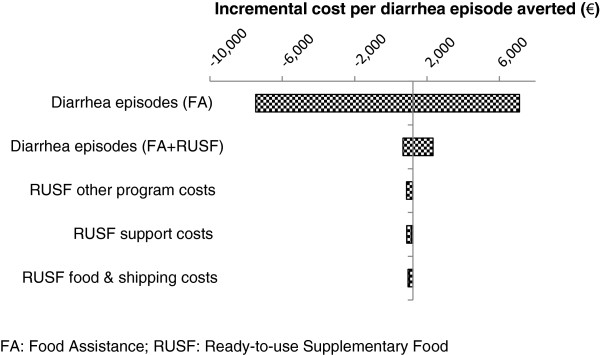
Tornado diagram: diarrhea outcome.

Figure 2 shows the model is most sensitive to changes in the number of diarrhea episodes per month for children in the FA program. When assuming lower levels of diarrhea incidence in the FA intervention area (i.e. higher effectiveness of FA alone in preventing diarrhea), FA alone dominated FA + RUSF, i.e. was more effective and less costly. This is indicated by a small incremental effectiveness and a resulting large negative ICER value (−7,472). Aside from this area of dominance, a 35% change in the episodes of diarrhea averted in the FA program resulted in ICER values ranging between one-half and nearly 7 times the base case (range: 485–7,116). There was no change of strategy indicated in the sensitivity analysis for effectiveness of FA + RUSF, suggesting that given a 25% change in the effectiveness of FA + RUSF, this strategy remained more cost-effective than FA alone for averting episodes of diarrhea.

Changes in cost variables had a relatively smaller effect on the ICER. If locally-produced RUSF were available, the ICER would decrease to 954 EUR in the best case, assuming a decrease in purchase price of local product and no charges for international shipping. Scenarios using both high and low cost estimates of purchase price of local RUSF resulted in a difference in outcomes of between 6% and 8% (range: 954–972 EUR). This indicates that in this program the purchase price of local product affected results less than the cost of international shipping (which was assumed to be zero in both scenarios). The proportion of support costs allocated to the program yielded a change of at most 15% (range: 883–1,151 EUR), indicating that support cost allocation did not have a strong influence on cost-effectiveness outcomes. A 25% change in all other program costs (excluding RUSF and support costs) resulted in a 16% change in the ICER (range: 872–1,203).

**Figure 3:**
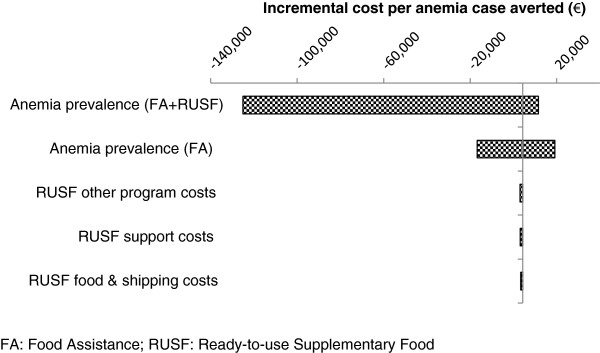
Tornado diagram: anemia outcome.

As Figure 3 demonstrates for the anemia outcome, the model was most sensitive to the difference in anemia prevalence between the FA and FA + RUSF interventions. When assuming higher prevalence of anemia in the FA + RUSF group, or lower prevalence in the group receiving FA alone (i.e. lower effectiveness of RUSF in preventing anemia), the FA + RUSF intervention was dominated by FA alone. This resulted in large negative ICER values for these variables. Aside from this area of dominance, a 25% change in cases of anemia averted by FA + RUSF resulted in ICER values between less than one-half to over 3 times the base case (range: 1,530-11,558). Similarly, a 25% change in cases of anemia averted in the FA program resulted in ICER values up to 5 times the base case (range: 1,384-19,159).

The model was less sensitive to changes in program costs. Assuming a reduction in purchase price of RUSF, and excluding international shipping costs, resulting ICERs were 6-8% less than the base-case (range: 3,336-3,399). Given a plausible range of support cost allocation, the ICER changed by at most 15% (range: 3,085-4,024), again indicating that support rate allocation did not strongly affect results. A plausible range (+/− 25%) of general program costs (excluding RUSF and support costs) resulted in a 16% change in results (range: 3,049-4,205).

### Probabilistic sensitivity analysis

**Figure 4:**
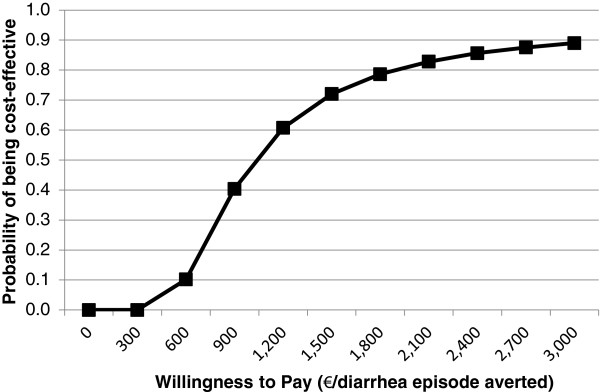
Cost-effectiveness acceptability curve for diarrhea outcome.

**Figure 5:**
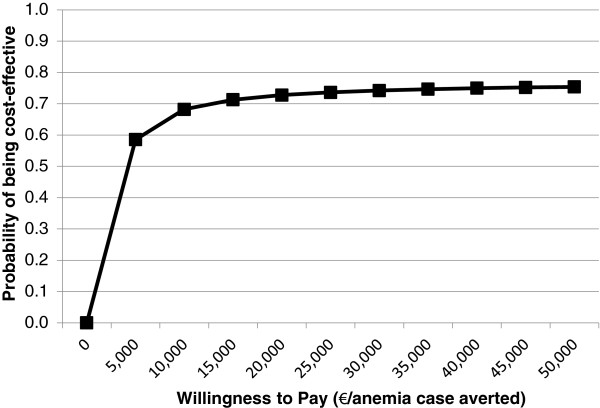
Cost-effectiveness acceptability curve for anemia outcome.

Figures 4 and 5 present results of probabilistic sensitivity analyses using cost-effectiveness acceptability curves representing cost per episode of diarrhea and case of anemia averted, from the societal perspective.

Acceptability curves show the probabilities that the FA + RUSF intervention would be cost-effective given a range of society’s hypothetical willingness to pay per case of anemia and episode of diarrhea averted. These curves demonstrate that the probabilities that the intervention would be cost-effective are 50% and 75% at a willingness to pay of 1,038 EUR and 1,634 EUR per episode of diarrhea averted, and 3,627 EUR and 40,709 EUR per case of anemia averted, respectively.

## Discussion

This analysis has demonstrated that, during the 2010 hunger gap in a Sahelian country, adding an RUSF supplement to a staple food ration distributed to vulnerable households resulted in an incremental cost per child of 374 EUR. The RUSF supplement, plus related management and logistics, cost an additional 1,083 EUR per episode of diarrhea averted and 3,627 EUR per case of anemia averted (hemoglobin <110 g/L).

Results from sensitivity analyses suggest that adding RUSF to a Food Assistance (FA) intervention would be considered cost-effective compared to FA alone, if society were willing to spend between approximately 1,000 and 2,000 EUR per episode of diarrhea averted, and between 4,000 and 40,000 EUR per case of anemia averted. Results were sensitive to assumptions about the effectiveness of FA + RUSF relative to FA alone.

The budget increase involved in adding RUSF to a general food distribution was 23%, which is less than the 34-52% increase forecast in other studies [4]. Using available estimates of purchase price for both imported and locally-produced RUTF, we estimated that if locally-produced RUSF were available in Chad and were used in this intervention instead of imported product, cost-effectiveness outcomes would have improved by 6-8%. Recent evidence has indicated that local production may not yield cost savings in all settings [45,46], and our analysis also found purchase price of local product to vary widely, from 1.89 EUR/kg in Malawi [44] to 3.39 EUR/kg in Niger (Nutriset, communication to ACF), compared to 2.83 EUR/kg for imported product. Given the relatively small quantity of RUSF consumed by the research cohort in this program, the international shipping cost had a larger effect on cost outcomes than purchase price of the product itself. Further research is needed to clarify the costs of local production, and whether this is a potential source of savings in supplementary feeding programs.

### Cost-effectiveness

#### Context of findings

While the addition of RUSF to a staple food ration did not result in a significant reduction in wasting rates [18], cost-effectiveness was assessed using the secondary outcomes of episodes of diarrhea and cases of anemia averted. Before further discussing findings of this cost-effectiveness study, it is necessary to specify their context and generalizability.

First, the lack of effect on wasting incidence of RUSF in this intervention adds to the inconclusive evidence around the effectiveness of LNS to protect child nutrition status [11-14,19]. One possible reason for this failure could be that wasting was related not to food insecurity—the context in which the LNS was designed to function—but to other factors in this urban setting such as poor hygiene and sanitation. Further, cost-effectiveness of LNS was assessed using secondary outcomes for which the product was not designed and may not be expected to perform well.

There continues to be interest in use of LNS to prevent malnutrition and morbidity, despite the lack of official recommendations and inconclusive evidence around its use for such purposes. Until the benefits of LNS used for preventive purposes have been proved more definitively, other options should be considered for protecting the nutrition and health of vulnerable populations in emergency settings. There is also need to further define which potential solutions and service delivery strategies are reliably effective and cost-effective in different settings.

This study contributes cost-effectiveness evidence from one operational research trial. While the cost-effectiveness of therapeutic use of LNS has been documented in various settings [47-50], we are aware of no other studies assessing the economic implications of preventive use of these products. With a dearth of comparable economic data, this discussion has sought to put this intervention strategy in the context of the costs and effectiveness of other interventions commonly employed to avert cases of anemia and diarrhea in young children. Not all of these interventions are directly comparable with ours due to differences in factors such as emergency context, limited public infrastructure, target population and breadth of intervention objectives.

#### Comparison with other research

##### Anemia prevention

Iron deficiency anemia (IDA) affects cognitive ability and work capacity, with evidence of long-term effects on mental and motor development in children under two years [51]. Its occurrence in children has many causes, including poor diet quality and blood loss due to intestinal helminths [52].

Children in Abeche receiving RUSF, compared to those in households receiving a staple ration alone, had blood hemoglobin concentrations that were higher by 3.8 g/L, and prevalence of anemia that was 10 percentage points lower. These results are within the range considered to be significant in a recent review on effectiveness of complementary feeding interventions [5]. This indicates that program effectiveness in terms of anemia outcomes compares favorably with other similar intervention strategies.

National iron fortification programs achieve great cost-efficiency in preventing anemia due to their ability to achieve high coverage, decreasing anemia rates by 10 to 20 percentage points in some populations [53]. One study estimated that an iron fortification program cost around US$ 1.33 per case of IDA prevented among school-aged Venezuelan children [51]. However, there is limited evidence of the ability of mass fortification of staple foods with iron to prevent anemia in pre-school aged children, since this age group has large iron requirements relative to their energy needs and typically consumes small amounts of staple foods [4].

Supplementing the diet with fortified complementary foods is an appropriate intervention to increase iron stores among preschool-aged children, typically requiring more personnel and supervision, and hence more resources than fortification programs [53,54]. While traditionally, micronutrient supplementation programs are considered less cost-effective than fortification programs−due in large part to the program structure requiring more intensive use of personnel [54]−both strategies are regarded by the WHO as being highly cost-effective, in that they avert a disability-adjusted life year (DALY) for less than three times a country’s per capita GDP [55].

Micronutrient sprinkles can be used for home fortification of complementary foods. One study modeling economic gains from use of sprinkles estimated a cost per child of US$ 1.20 including sprinkles sachets, distribution and overhead [56]; this is significantly lower than the cost per child in Chad. While the present analysis provides more comprehensive cost estimates, and takes place in an emergency setting with high logistics costs, it is likely that given the equal or greater effect of sprinkles on hemoglobin concentration and anemia prevalence [57], that the cost-effectiveness of sprinkles would be greater than that of LNS in averting cases of anemia. However, a comparison of sprinkles and LNS on this outcome alone would not capture the additional benefits provided by the increased energy, protein, and fatty acid content of LNS compared to sprinkles—which are used to enhance the micronutrient content, but not the macronutrient content of locally-available complementary foods—thereby underestimating its overall effectiveness and cost-effectiveness. A more appropriate comparative study would need to account for multiple outcomes including linear growth, for which micronutrient supplementation alone has not proven effective in several studies [57-60].

Prior research has assessed the cost-effectiveness of school-based helminth control interventions to avert anemia; these programs typically carry low fixed costs as children are treated in their own schools. A national school-based helminth control program run by the Ministry of Health in Uganda achieved a cost per case of anemia averted of US$ 3.19 [61]. An assessment of a school-based anthelmintic program in the Tanga Region of Tanzania estimated a cost per case of moderate anemia (hemoglobin <110 g/L) prevented of US$ 7.23 [62]. In an assessment of a school-based helminth control program in Zanzibar providing three doses of mebendazole per year, Stoltzfus et al. [63] estimated a cost of US$ 3.57 per case of moderate anemia averted.

This comparison of costs for different interventions addressing anemia does not account for important differences among these interventions. For example, supplementary feeding programs and school-based helminth control programs target different age groups (school-aged versus preschool-aged children), and a different range of outcomes. Further, these programs require functional public infrastructure, which may not be available in an emergency context.

##### Diarrhea prevention

Diarrhea is one of the leading causes of death among children [64], and can be caused by a variety of infections, including bacteria, viruses and protozoa [65]. Choice of intervention to prevent diarrhea may focus either on reducing exposure to pathogens, i.e. via improved water and sanitation, or on increasing resistance to infection, e.g. through micronutrient supplementation or promotion of breastfeeding, depending on the targeted age group and primary cause of infection in a particular setting. A recent review found limited and inconsistent evidence of the impact of complementary feeding on diarrhea incidence and prevalence [5].

There is also little evidence about the cost-effectiveness of child feeding programs to reduce or prevent episodes of diarrhea. An early study by the World Health Organization (WHO) reviewed the evidence at the time around potential cost-effectiveness of using supplementary feeding programs (SFPs) to prevent diarrheal disease, finding these interventions to not be cost-effective due to their high costs, along with the high levels of management and logistics which they required [66]. This review instead recommended other interventions with more potential to be cost-effective at diarrhea prevention, including immunization against rotavirus, cholera and measles infection; improvements in water supply, sanitation and hygiene; and promoting improved breastfeeding and weaning practices [65].

While early research did not support use of supplementary feeding for prevention of diarrhea, these investigations were conducted before the development of LNS. Considering the proven role of micronutrients, particularly zinc, in preventing and treating diarrhea [56,67,68], the enhanced micronutrient profile of these supplements may make SFPs more effective, and therefore more cost-effective for this purpose. However, despite advancements in food technology, distribution programs carry high costs for food (both rations and RUSF in the case of the Chad program), along with its management and logistics. It is difficult for programs with such a cost structure to achieve cost-effectiveness outcomes comparable with other programs which do not include costly food items.

One study in Latin America determined that breastfeeding promotion cost between US$ 0.65 and US$ 6.75 per case of diarrhea averted among infants [69]. This finding highlights the need to ensure that breast milk is not replaced by LNS in supplementary feeding programs [4]. However, such programs are targeted to children older than 6 months, who should consume complementary foods in addition to breast milk. To prevent diarrhea in this age group, it is therefore critical to address the quality and safety of these complementary foods.

Use of locally available complementary foods is the preferred approach to improve nutrient status in the longer-term, although it is recognized that it is difficult to meet dietary requirements to prevent iron deficiency where diets are of poor quality and mostly plant-based, and where there is inadequate diet diversity [70,71]. Cost-effectiveness evidence on food-based approaches is needed, although in the majority of settings where such approaches are feasible, they are likely to be preferred to use of LNS based on sustainability concerns among others.

Water, sanitation and hygiene interventions are often implemented to prevent diarrheal morbidity in areas with poor hygiene. One study assessing the potential cost-effectiveness of a population-level water supply and sanitation intervention demonstrated that oral rehydration solution plus hygiene education would cost US$ 2.93 per episode of diarrhea averted [72]. Another study, on a home-based chlorination and safe water storage intervention to reduce diarrhea among people living with HIV in rural Uganda, estimated a cost of US$ 5.21 per episode of diarrhea averted [73]. A study in Burkina Faso analyzed the cost-effectiveness of a large-scale urban hygiene promotion program, finding an incremental cost of US$ 51.30 per case of child diarrhea prevented from the societal perspective [74].

Many of these favorable cost-effectiveness findings were dependent on the existence of physical water and sanitation infrastructure. In Abeche where the ACF program took place, poor hygiene was a major issue affecting child health, which suggests that the existing water and sanitation infrastructure may have been inadequate. Although the Chad program started with a Food for Training element focusing on hygiene promotion, this intervention component was discontinued due to security issues limiting the movement of field staff. This highlights the underlying difficulties in implementing such programs in insecure settings.

#### Implications

Given limited data on cost-effectiveness of preventive use of LNS, this discussion has sought to contextualize the costs and effectiveness of this intervention strategy among others typically implemented to avert cases of anemia and diarrhea in young children. As stated above, results from these other studies are not directly comparable with the present study, for several key reasons related to intervention context and objective.

Emergency settings limit the range of interventions which it is feasible to deliver to a population. School-based programs, for example, may require a relatively stable atmosphere in which to be conducted effectively. Further, school-based disease control interventions have a very different cost structure from an NGO-run emergency food distribution carrying costs of staple food rations and LNS. It is unlikely that a food distribution program would achieve similar cost-effectiveness results as a low-cost vertical program addressing a specific illness. National-level programs such as school-based helminth control and iron fortification carry the potential to have among the lowest costs and highest effectiveness of any program addressing child health, assuming that there exists such infrastructure with adequate capacity in-country. It is also possible that programs implemented by functioning government bodies, with greater economies of scale, hold more potential to achieve higher levels of cost-effectiveness given the larger infrastructure and broader human resource base to which they have access, compared to an NGO program, particularly in an emergency setting. Given that Chad’s 2010 per capita GDP was 226 EUR [75], only the most cost-effective programs would be advisable for implementation by government agencies working within these limited budget parameters.

Further, all “emergency” settings are not the same. Hunger gaps in Chad occur on a regular basis, and there are many contextual factors, including political instability and food insecurity, which contribute to the chronic vulnerability of the population in this study.

Given the levels of acute malnutrition seen in the population in the months leading up to the program, there was clear justification for intervention. However, the ultimate value to the community of a 5-month one-off distribution program within this vulnerable context is questionable. The attrition seen mid-intervention among recipients of FA alone was eventually corrected through use of incentives. While this reflects an incipient humanitarian dependency in this relatively under-served population, it also signals, to some extent, the community’s valuation of the program. A needs assessment conducted during program planning found that poor hygiene and sanitation were major factors contributing to poor health and malnutrition in this urban setting. These elements of the program were eventually too cumbersome to implement due to insecurity and were discontinued.

There is a need to think strategically about what kinds of interventions would have the best chance for useful, sustained impact in this and similar settings. There is evidence that food assistance programs used in the longer-term in a preventive manner can be more effective than curative programs for improving child nutrition status [76]. Such an ongoing preventive program would be better-equipped to address the structural causes of malnutrition, and could be linked to a flexible rapid-response mechanism during the inevitable hunger gap.

General food rations distributed in emergencies include cereals, pulses, a fortified blended food such as CSB, oil, salt and sugar; and research indicates that these rations do not meet the nutritional needs of infants and young children [4]. There is thus a need for an improved formula. Addition of a lipid nutrient supplement (LNS) to a general food distribution ration, as was done in this study, is one option for improving the nutrition profile of these rations to meet the needs of vulnerable population groups.

LNS are specially manufactured foods, requiring financial outlays for local and usually international shipment, and carry a high cost relative to other food options for international nutrition programs. Cost of the RUSF was a small proportion of total costs in this study. This is because the amount of RUSF used for this research cohort (FA + RUSF only) was relatively small. In a program setting with full population coverage, expected cost of product (and related transportation) would be much higher. LNS has comprised between 20 and 40% of total program costs when used for therapeutic purposes [47-50]. Though the amount consumed per child for supplementary feeding would be lower compared to therapeutic feeding, there is no evidence as yet of the actual cost of using supplementary LNS at scale.

There exist other options for improving nutritional quality of supplementary foods. A recent Cochrane review concluded that CSB++ may be equally effective and cheaper than LNS for treatment of moderate wasting [77]. This review also found a lack of studies to improve the quality of the home diet in settings where this is feasible, suggesting that this potential has not been adequately explored in the context of preventing and treating moderate wasting. The WHO, WFP and UNICEF have recommended a daily multiple micronutrient supplement formula to deliver to vulnerable groups during emergencies, including young children and pregnant and lactating women [78]. Micronutrient sprinkles have also been found to be feasible for use in emergency settings [79], although these strategies focusing on micronutrients alone do not improve the macronutrient content of local foods as discussed previously. There is also potential for reducing acute wasting incidence through other service delivery strategies, including cash transfers, hygiene programs and behavior change interventions.

Given the alternatives available, and the conflicting evidence on the effectiveness of LNS when used in a supplementary form, there is need for research to define what strategies work best in delivering adequate nutrition to vulnerable populations in emergency settings, and in what contexts these strategies can be relied upon to achieve positive outcomes in protecting child health and nutrition status. The evidence shows that we do not yet have clear answers to these questions.

### Limitations

This study has several limitations which may have influenced cost-effectiveness findings. First, this intervention produced several potential positive outcomes, the sum of which should be considered the full program outcome; this study has only assessed costs per individual disease outcomes. Next, diarrhea and anemia were secondary outcomes of the RCT and may not be the best indicators to reflect the effectiveness or cost-effectiveness of RUSF supplementation; however morbidity outcomes are commonly assessed in LNS studies [6,14-17,21,23]. Additionally, diarrhea was measured by recall, which although common practice [16] may not have been sufficiently accurate. Lastly, the short duration of the study may not have been adequate to see the effects intended, particularly considering the contextual and structural causes of malnutrition in Abeche.

In terms of the anemia outcome, additional measures could have been taken to ensure that children ingested and absorbed all the nutrients needed to make red blood cells. As we know neither the proportion of RUSF actually consumed (these analyses are still underway), nor the proportion of these nutrients absorbed in this setting, it is possible that RUSF consumption in this study was not optimized to prevent anemia. However, these measures would have been appropriate for an efficacy trial, whereas this effectiveness study aimed to measure outcomes in a pragmatic setting. Finally, given the poor sanitation in Abeche, it would have been preferable to control for parasitic infections in this population, although Food for Training sessions focusing on hygiene were initiated but not fully implemented due to insecurity in the area.

Regarding the cost assessment, it was not possible to interview all program staff regarding their time allocation. Moreover, due to the time elapsed since the program was implemented, it may have been difficult for key informants to recall with accuracy their time allocation, which would challenge the accuracy of results. However, given our access to key staff with in-depth knowledge of the program, we do not feel that these potential constraints diminish the findings.

### Future research

Despite these limitations, our analysis was the first to contribute findings on the cost-effectiveness of preventive use of LNS, and provides insights into future areas of research. There is a need for definitive information on the local production of LNS, including cost and feasibility of timely delivery and standardized quality. A thorough costing study should be conducted to determine the total costs of local production, including costs for quality assurance measures and discussion of effect on costs of market size and scale of production, to determine whether there are potential cost savings in scaling up local production. Additionally, research should be conducted to compare an operation by an international NGO with a similar program run by governments and other local stakeholders to assess potential differences in levels of effectiveness and cost-effectiveness achievable by each implementing agency in the same setting. This would provide a basis for determining the most appropriate and cost-effective service delivery mechanism for various interventions. This study assessed cost-effectiveness retrospectively; future RCTs conducted in emergency settings should take the opportunity to document costs of ongoing research interventions. Such studies would need to consider how, in an emergency setting, to collect cost data that is to be used for analytical purposes, including both careful documentation of costs, and potentially measuring staff time allocation for activity-based costing. Further consideration needs to be given in such settings to how to achieve the best tradeoff between methodological rigor and field realities. Finally, given inconclusive evidence, further research is warranted to establish in which contexts LNS is most effective and cost-effective to protect child health and nutrition status, compared to other alternatives, including micronutrient supplements, improved local complementary foods, behavior change interventions, cash transfers and hygiene programs as a few examples.

## Conclusions

While the addition of RUSF to a staple ration distribution did not prevent cases of wasting in young children, we assessed cost-effectiveness using secondary outcomes of diarrhea and anemia cases averted. The cost-effectiveness of this approach was poor when compared to other common intervention strategies. While food-based programs, such as ration distribution or supplementary feeding with RUSF, may not be among the most cost-effective solutions to child morbidity, these interventions play an important role in preserving food security, livelihoods and nutritional status among vulnerable adults and children. Further, RUSF holds the potential to address multiple health and nutrition outcomes in emergency contexts, making it a promising short-term option to protect child health and nutrition in settings where diets are poor and public health infrastructure is weak. However, given inconclusive evidence, further research is needed to determine the contexts in which RUSF is most effective and cost-effective to protect child health and nutrition status, compared to other alternatives.

## Competing interests

The authors declare that they have no competing interests.

## Authors’ contributions

CP participated in study design, collected, analyzed and interpreted data and drafted the manuscript. CS participated in study design and coordination, contributed to data interpretation and was involved in drafting the manuscript. EL and FH contributed to data acquisition and interpretation, and helped to revise the manuscript. MA and AI participated in study design and coordination, and helped to revise the manuscript. All authors read and approved the final manuscript.
